# Interactions between ecological factors in the developmental environment modulate pupal and adult traits in a polyphagous fly

**DOI:** 10.1002/ece3.5206

**Published:** 2019-04-26

**Authors:** Binh Nguyen, Fleur Ponton, Anh Than, Phillip W. Taylor, Toni Chapman, Juliano Morimoto

**Affiliations:** ^1^ Department of Biological Sciences Macquarie University North Ryde New South Wales Australia; ^2^ Department of Entomology Vietnam National University of Agriculture Hanoi Vietnam; ^3^ New South Wales Department of Primary Industries The Elizabeth Macarthur Agricultural Institute Meneagle New South Wales Australia

**Keywords:** animal–microbe competition, crowding, density, larval competition, microbiota

## Abstract

1. In holometabolous insects, adult fitness depends on the quantity and quality of resource acquired at the larval stage. Diverse ecological factors can influence larval resource acquisition, but little is known about how these factors in the larval environment interact to modulate larval development and adult traits.
2. Here, we addressed this gap by considering how key ecological factors of larval density, diet nutritional composition, and microbial growth interact to modulate pupal and adult traits in a polyphagous tephritid fruit fly, *Bactrocera tryoni* (aka “Queensland fruit fly”).
3. Larvae were allowed to develop at two larval densities (low and high), on diets that were protein‐rich, standard, or sugar‐rich and prepared with or without preservatives to inhibit or encourage microbial growth, respectively.
4. Percentage of adult emergence and adult sex ratio were not affected by the interaction between diet composition, larval density, and preservative treatments, although low preservative content increased adult emergence in sugar‐rich diets but decreased adult emergence in protein‐rich and standard diets.
5. Pupal weight, male and female adult dry weight, and female (but not male) body energetic reserves were affected by a strong three‐way interaction between diet composition, larval density, and preservative treatment, whereby in general, low preservative content increased pupal weight and female lipid storage in sugar‐rich diets particularly at low‐larval density and differentially modulated the decrease in adult body weight caused by larval density across diets.
6. Our findings provide insights into the ecological factors modulating larval development of a polyphagous fly species and shed light into the ecological complexity of the larval developmental environment in frugivorous insects.

## 1 INTRODUCTION

Resources acquired at the early stages of development determine the fitness of adults and their offspring (Rowe & Houle, 1996). In holometabolous insects, key ecological factors such as larval density, diet composition, and the microbial community colonizing the diet can modulate larval nutrition and, in turn, influence adult reproductive success, offspring quality, and the survival of groups and populations (Drew & Lloyd, 1989; Fitt & O'Brien, 1985; Morimoto et al., 2019; Sentinella, Crean, & Bonduriansky, 2013; Storer, Wainhouse, & Speight, 1997). While the implications of these ecological factors have been investigated individually or in pairs across many species, challenges remain in better understanding the combined effects of diverse ecological factors in shaping the larval environment (Wertheim, Marchais, Vet, & Dicke, 2002).

Larvae of many insects tend to aggregate in high density (Durisko & Dukas, 2013; Ives, 1991; Taylor, 1961; Taylor, Woiwod, & Perry, 1978) often with positive effects on individual fitness across taxa, including Diptera, Coleoptera, and Lepidoptera (the “Allee effect” [Allee, Park, Emerson, Park, & Schmidt, 1949; Courchamp, Clutton‐Brock, & Grenfell, 1999]; see e.g., Appleby & Credland, 2007; Lawrence, 1990; Morimoto, Nguyen, Tarahi Tabrizi, Ponton, & Taylor, 2018; Weaver & Mcfarlane, 1990). Despite this, the positive effects of aggregation depend on diet composition. Nutrient‐poor larval diets created by high larval densities have been shown to delay pupation, increase pupal mortality, and result in small adult body size (Gage, 1995; Morimoto, Pizzari, & Wigby, 2016; Stockley & Seal, 2001). This is likely because high larval density decreases the availability of protein (and consequently amino acids) to the developing larvae (Klepsatel, Procházka, & Gáliková, 2018). If larvae are foraging in nutrient‐poor diets, the costs of nutrient limitation and competition in larval aggregations can be high and rapidly offset the benefits of aggregation, and thus, larval aggregation patterns tend to be diet‐dependent (Morimoto et al., 2018). However, diet quality is defined not only by its nutritional composition but also by its microbial community. This is because microbes in the diet can modulate larval growth by modifying the diet composition, being a direct source of nutrients to the larvae and, in some cases, replenishing the host gut flora (Drew, 1988; Drew, Courtice, & Teakle, 1983; Matavelli, Carvalho, Martins, & Mirth, 2015; Wong et al., 2017). For instance, microbes in the diet increase amino acid availability for tephritid fruit fly larvae (Drew, 1988) while *D. melanogaster* are attracted to diets with microbes that match their own gut microbiota community (Drew, 1988; Wong et al., 2017). In *Bactrocera tryoni,* adults feed on microbes to supplement their nutrition (Drew et al., 1983). Microbes in the diet also influence foraging behavior by releasing odors that attract larvae and gravid females searching for oviposition sites (Durisko & Dukas, 2013; Venu, Durisko, Xu, & Dukas, 2014; Wertheim et al., 2002; Wong et al., 2017), which in turn might influence the density of larvae foraging in a patch at a given time. Thus, the network of interactions between larval density, diet composition, and microbial growth in the diet is complex and certainly shapes larval development. No direct empirical test addressing this complexity has yet been performed, and key questions remain, such as “Can microbial growth in the larval environment mitigate (or accentuate) density‐ and diet composition‐dependent effects on larval development?”; “How does the three‐way interaction between larval density, diet composition, and microbial growth affect fitness‐related traits of individuals?”.

To address these questions, we manipulated larval density (“low” and “high”), larval diet composition through manipulating the ratio of dietary yeast and sugar (Y:S ratio), and preservative content (“low” and “high”) in the larval environment of a polyphagous fruit fly pest, *Bactrocera tryoni* (Froggatt) (Diptera: Tephritidae; aka “Queensland fruit fly”). Low preservative encouraged microbial growth in the diet (Figure S1) and was designed to simulate microbial growth experienced in ripening and decaying substrates commonly experienced by fly larvae (e.g., Matavelli et al., 2015). We tested our predictions arising from previous literature on the single and interactive effects of these three factors on pupal weight, adult emergence, adult body mass, and adult energetic reserves (lipid storage) (see Table 1 for predictions). The wide variety of hosts that are exploited by *B. tryoni* (i.e., 117 hosts known so far; Clarke, Powell, Weldon, & Taylor, 2011) and its status as an effective invader that can readily expand its host range (see [Clarke et al., 2005; Clarke et al., 2011; Vargas, Pinero, & Leblanc, 2015]) makes this species an important target for better understanding how the complex ecological interactions in the larval developmental environment contributes to developmental and adult traits. This study adopts an integrative approach to explore the combined effects of key ecological factors shaping the larval environment. By understanding how larval density, diet composition, and microbial growth interact, our findings provide insights into the ecological factors modulating the ontogeny of many frugivorous insect species.

**Table 1:** Hypothesis and predictions tested in this study

Hypothesis	Predictions	References
High larval density is costly	1. Low pupal weight in high larval densities; 2. Low adult weight in high larval densities	Bauerfeind and Fischer (2005), Gage (1995), Morimoto et al. (2016), Stockley and Seal (2001)
High larval density induces nutrient‐poor phenotypes that are rescued by microbial growth	3. Relatively minor effects of high larval density on pupal and adult traits when larvae feed on protein‐rich diets and/or diets where microbial growth was encouraged in low preservative content diets, because microbes could serve as surplus of protein to the larvae	Klepsatel et al. (2018)
Protein is an essential nutrient for adequate larval development	4. High adult emergence in protein‐rich diets	Kaspi, Mossinson, Drezner, Kamensky, and Yuval (2002), Nestel and Nemny‐Lavy (2008), Rodrigues et al. (2015), Silva‐Soares et al. (2017)
Sugar‐rich diets during development increase lipid storage	5. High pupal weight in sugar‐rich diets; 6. High percentage of lipid storage for adults raised in sugar‐rich diets	Musselman et al. (2011), Na et al. (2013), Nestel, Nemny‐Lavy, and Chang (2004)
Microbial growth modifies nutrient composition and serves as nutrient source for the larvae	7. Larvae fed on protein‐rich and sugar‐rich diets in which microbial growth was encouraged due to low preservative content to have lower body mass than larvae fed on diets in which microbial growth was inhibited due to high preservative content. In particular, larvae fed on protein‐rich diets with low preservative content could be leaner and have the lowest pupal and adult weights (Figure 1)	Drew et al. (1983), Fitt and O'Brien (1985)

**Figure 1:**
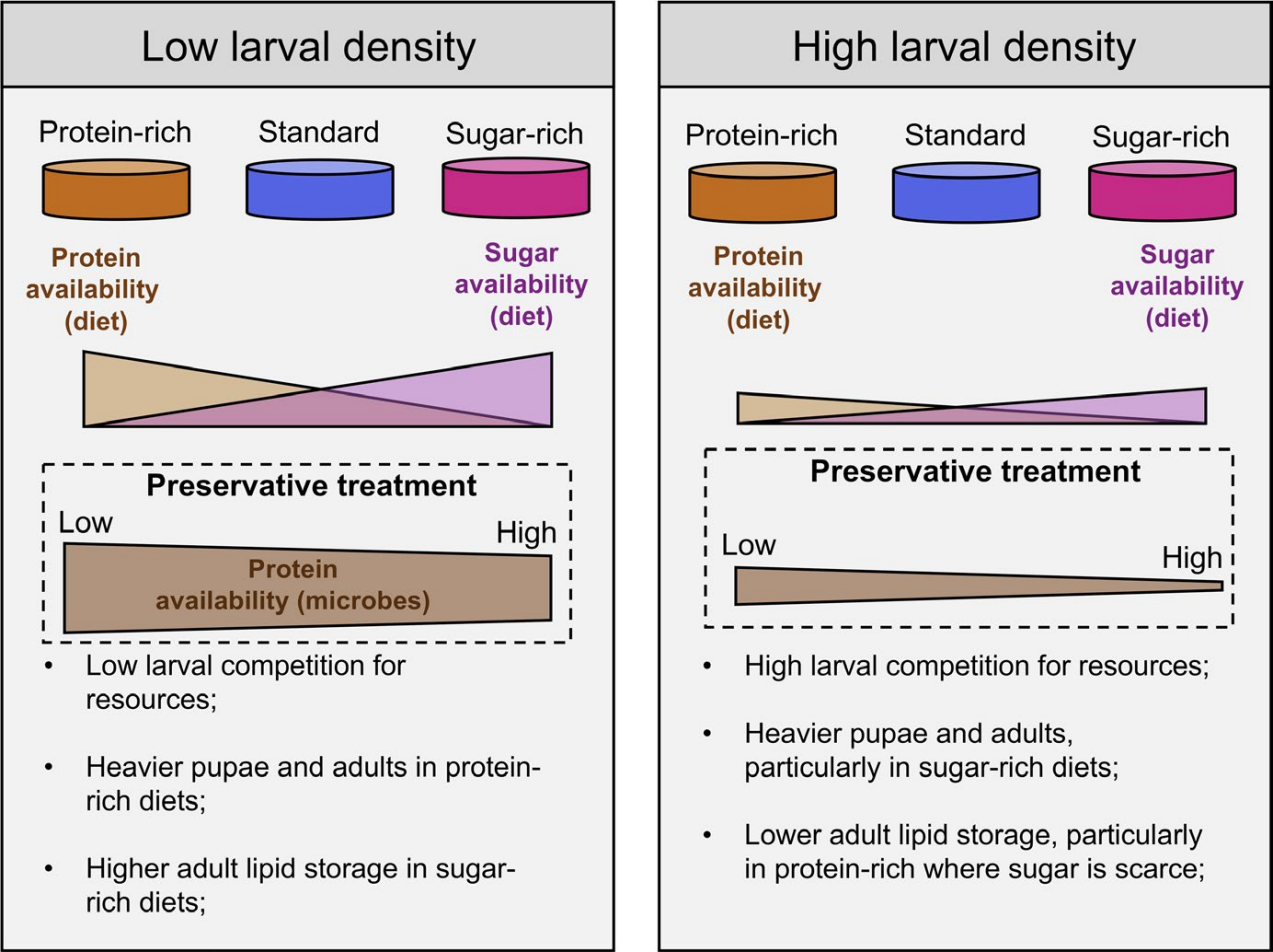
Overview of the experimental design and predictions. Microbial growth arising from low preservative content in the diet was expected to supplement the availability of protein to the larvae. High larval density was expected to reduce overall availability of nutrients (both protein and sugar) as a result of increased larval competition. Orange—protein‐rich diet (Y:S ratio 4:1); Blue—standard gel‐based diet (Y:S ratio 1.6:1); Magenta—sugar‐rich diet (Y:S ratio 1:2)

## 2 MATERIALS AND METHODS

### 2.1 Fly stock

Eggs were collected from females in our laboratory‐adapted stock of *B. tryoni* that was established in 2015 (>20 generations old). The stock has been maintained in nonoverlapping generations, in which adults were provided a free‐choice diet of hydrolyzed yeast (MP Biomedicals Cat. n^o^ 02103304) and commercial refined cane sugar (CSR^®^ White Sugar), while larvae were maintained for the last 10 generations using a gel diet formulation (Moadeli, Taylor, & Ponton, 2017) that is based on a liquid diet formulation of Chang, Vargas, Caceres, Jang, and Cho (2006). All stocks and experiments were maintained in a controlled environment room at 65 ± 5% relative humidity and 25 ± 0.5°C, with light cycles of 12 hr light: 0.5 hr dusk:11 hr dark: 0.5 hr dawn in the Department of Biological Sciences at Macquarie University.

### 2.2 Experimental diets

We used three diets that varied in yeast‐to‐sugar ratio (i.e., Y:S ratio). The “standard diet” followed the gel‐based diet recipe of Moadeli et al. (2017) (see Table S1). We then used the same recipe but modified the amount of yeast and sugar to create a “protein‐rich diet” (Y:S ratio 4:1) and a “sugar‐rich diet” (Y:S ratio 1:2) (see Table S1 for recipe). When included (see “Experimental design” below), the preservatives were Nipagin (Southern Biological^®^ cat no. MC11.2) and Sodium Benzoate (Sigma^®^ cat no. 18106). Note that although Citric Acid (Sigma^®^ cat no. C0759) is a preservative (Davidson, Taylor, & Schmidt, 2013), it is also needed to control the pH of the media and therefore was used in the diets as recommended in the original recipe (see Moadeli et al., 2017). Hence, we had two preservative treatments: low and high. There were notable differences in microbial growth on diets with low and high preservatives contents (Figure S1), which supports our assumption that preservative treatments manipulated primarily microbial growth in the diets. In total, we had twelve treatments (three diets × two densities × two preservative treatments). For treatments where preservative content was high, we had *15* replicates per larval density per diet; for treatments where preservative content was low thereby encouraging microbial growth, we had *5* replicates per larval density per diet.

### 2.3 Larval rearing

To generate low and high larval densities, 10 and 40 μl of egg–water solution in a final concentration of ca. 16 eggs/ml of diet and 62 eggs/ml of diet, respectively, were placed in 90 mm Petri dishes containing 15 ml of each of the experimental diets, and the Petri dishes were covered. After seven days, when third instar larvae were ready to exit the diet to pupate, the lids were removed and the uncovered Petri dishes were placed into larger plastic containers (16 cm × 14.3 cm × 14.5 cm) that contained ca. 50 g of fine vermiculite for pupation. Pupae were sifted from the vermiculite two to three days after the dishes were placed onto the vermiculite and were transferred to a 90 mm Petri dish for weighing. Next, 40 pupae per replicate per diet composition per preservative treatment per larval density were then transferred in an open 50‐ml Petri dish to a 5 L plastic container until adults emerged. Upon emergence, adults were provided water and a free‐choice diet of hydrolyzed yeast (MP Biomedicals Cat. n^o^ 02103304) and commercial refined cane sugar (CSR^®^ White Sugar) for three days prior to our assessment of body lipid storage. All flies had unlimited access to water throughout the experiments.

### 2.4 Pupal weight and adult percentage of emergence

Pupal weight was assessed by weighing 12–15 randomly selected pupae per replicate per diet per larval density per preservative treatment (*N* = 1620) on a Sartorius^®^ ME5 scale (0.0001 g precision). All pupae were weighed seven days after pupation. Percentage of adult emergence was assessed by counting the number of adults that emerged, dividing by the total number of pupae (i.e., 40) and multiplying by 100 (%).

### 2.5 Lipid storage (energetic reserves) quantification

Four to eight three‐day‐old males and four to eight three‐day‐old females per replicate per diet composition per larval density per preservative treatments (*N = *300 males and 300 females) were placed individually in 10‐ml glass tubes, freeze‐killed (−20°C), and dried at 60°C for three days in a drying oven. Dried bodies were weighed on a Sartorius^®^ ME5 scale (0.0001 g precision). To extract lipids (Ponton et al., 2015), two mL of chloroform (Sigma Aldrich^®^, Cat no. 288306) was then added to each tube which was then sealed with a rubber plug and held for 24 hr before the chloroform was discarded. The chloroform lipid extraction procedure was repeated three times on consecutive days. Bodies were then dried again at 60°C for three days in a drying oven before we measured body weight after lipid extraction. The percentage of body lipid was calculated as the difference between the dry body weight before and after lipid extraction, standardized by the body weight of each fly before the lipid extraction multiplied by 100 (i.e., percentage of lipid relative to the original dry body weight of each fly).

### 2.6 Statistical analyses

All statistical models evaluated the statistical significance of main and interactive effects. We did not exclude nonsignificant interactions because interactions were part of our a priori predictions and therefore needed to be included in the final model. We nonetheless provide the final models of model selection approach in the Supplementary Information (see Tables S2–S6). Note that statistical inferences using model selection or full models converged to the same qualitative results, which corroborates the robustness of our full‐model approach. Assumptions of the models were assessed using inbuilt diagnostic plots in the statistical software. The statistical significance of larval density, diet composition, and preservative treatment on pupal weight and adult weight were examined using a generalized linear model (GLM) with Gaussian error distribution as this was the model that best fitted the data (Table 2). The statistical significance of larval density, diet composition, and preservative treatment on the percentage of adult emergence and percentage of body lipid, which are proportion data, were performed using a GLM with binomial error distribution and *quasi‐*extension to control for overdispersion of the data (Table 2). To control for pseudoreplication on the analysis of pupal weight, body weight, and percentage of body lipid, we used the average value per replicate (i.e., within replicate average) as the response variable and included replicate as a covariate in all models (see Table 2). P‐values were obtained from *F*‐statistics for all GLM models. All analyses were performed in R (R Development Core Team, 2017), and all plots were done using the “ggplot2” package (Wickham, 2009).

**Table 2:** Details of the statistical models used in this study

Dependent variable	Independent variables in the generalized linear model (GLMs)	Error distribution
Average pupal weight	~*larval density* * *diet composition* * *preservative treatment*	Gaussian
Percentage of adult emergence and sex ratio		quasibinomial
Average body weight^a^		Gaussian
Average percentage of lipid stored^a^		quasibinomial

a Sexes analyzed separately.

## 3 RESULTS

### 3.1 Larval density, diet composition, and preservative content interact to modulate pupal weight

**Figure 2:**
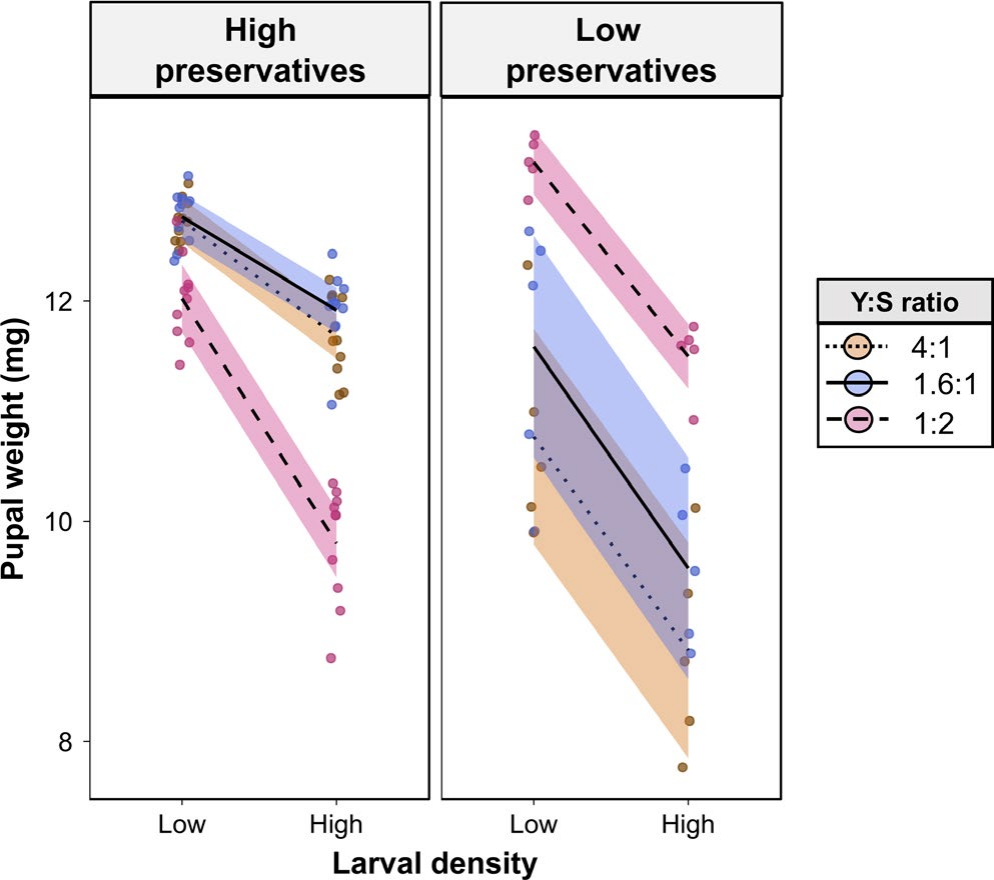
Interaction between larval density, diet, and preservative content on pupal weight. Given in mg. Lines were plotted using the ggplot2 package to guide interpretation of the results. Orange—protein‐rich diet (Y:S ratio 4:1); Blue—standard gel‐based diet (Y:S ratio 1.6:1); Magenta—sugar‐rich diet (Y:S ratio 1:2). “High preservatives”—diets with low preservative content where microbial growth was inhibited; “Low preservatives”—diets with low preservative content where microbial growth was encouraged. Points were “jittered” horizontally to avoid overlapping. Solid lines were drawn with the “loess” method from the “ggplot2” package to highlight trends in the data

There was a significant three‐way interaction between larval density, diet composition, and preservative treatment on pupal weight (*F*_2,78_ = 4.297, *p* = 0.017, Table S3); a decrease in pupal weight between low and high density varied with diet composition, and low preservative content affected pupal weight differently depending on both diet composition and larval density. Specifically, pupal weight was higher in protein‐rich and standard diet and lower in sugar‐rich diet when preservative content was high but the opposite was observed when preservative content was low (Figure 2).

On average, pupal weight was highest when larvae were reared at low density on the sugar‐rich diet when preservative content was low [Mean (*SD*): 13.26 (0.989)], and the lowest when larvae were reared at high density on the sugar‐rich diet when preservative content was low [Mean (*SD*): 8.82 (1.190)]. It is important to note that the difference in pupal weight between low and high larval density was more accentuated in the sugar‐rich diet when preservative content was high (Figure 2). This effect was largely due to a stronger decrease in pupal weight from low to high larval density in standard and protein‐rich diets when preservative content was low compared with the same decrease in sugar‐rich diet when preservative content was low (Figure 2). There were also statistically significant two‐way interactions between larval density and diet composition, larval density and preservative treatment, and diet composition and preservative treatment, as well as the main effects of larval density, diet composition, and preservative treatment (Table S3).

### 3.2 Diet composition and preservative content, but not larval density influence adult emergence

The three‐way interaction between larval density, diet composition, and preservative treatment was not statistically significant (*F*_1,108_ = 0.485, *p* = 0.616, Table S4). However, there was a significant interaction between diet composition and preservative treatment on the percentage of adult emergence (*F*_2,110_ = 4.729, *p* = 0.010, Table S4). This effect was driven by a decrease in the percentage of adult emergence in protein‐rich and standard diets but a sharp increase in sugar‐rich diet when preservative content was low compared with when preservative content was high (Figure S2). There were no significant main effects of larval density or preservative treatment on the percentage of adult emergence, although there was a significant effect of diet composition (*F*_1,116_ = 5.127, *p* = 0.007, Table S4) whereby sugar‐rich diets had on average lower percentage of adult emergence (Figure S2). There were no significant interactions between larval density and diet composition or larval density and preservative treatment (Table S4). There were also no effects of larval density, diet composition, preservative treatment, or any interactions among these factors, on the sex ratio of emerged adults (Table S4).

### 3.3 The interaction between larval density, diet composition and preservative content modulates adult body weight

There were statistically significant three‐way interactions between larval density, diet composition, and preservative content on female (*F*_2,48_ = 3.883, *p* = 0.027, Table S5) and male (*F*_2,48_ = 3.819, *p* = 0.028, Table S5) adult dry body weight. There was also a significant two‐way interaction between larval density and preservative treatment (*F*_1,52_ = 14.190, *p* < 0.001, Table S5), as well as the main effect of larval density (*F*_1,58_ = 23.537, *p* < 0.001, Table S5), preservative treatment (*F*_1,55_ = 5.845, *p* = 0.019, Table S5) for females, and a weak but significant two‐way interaction between larval density and preservative treatment (*F*_1,52_ = 4.190, *p* = 0.046, Table S5), as well as the main effects of larval density (*F*_1,58_ = 11.201, *p* = 0.001, Table S5) and diet composition (*F*_1,57_ = 4.944, *p* = 0.011, Table S5) for males.

**Figure 3:**
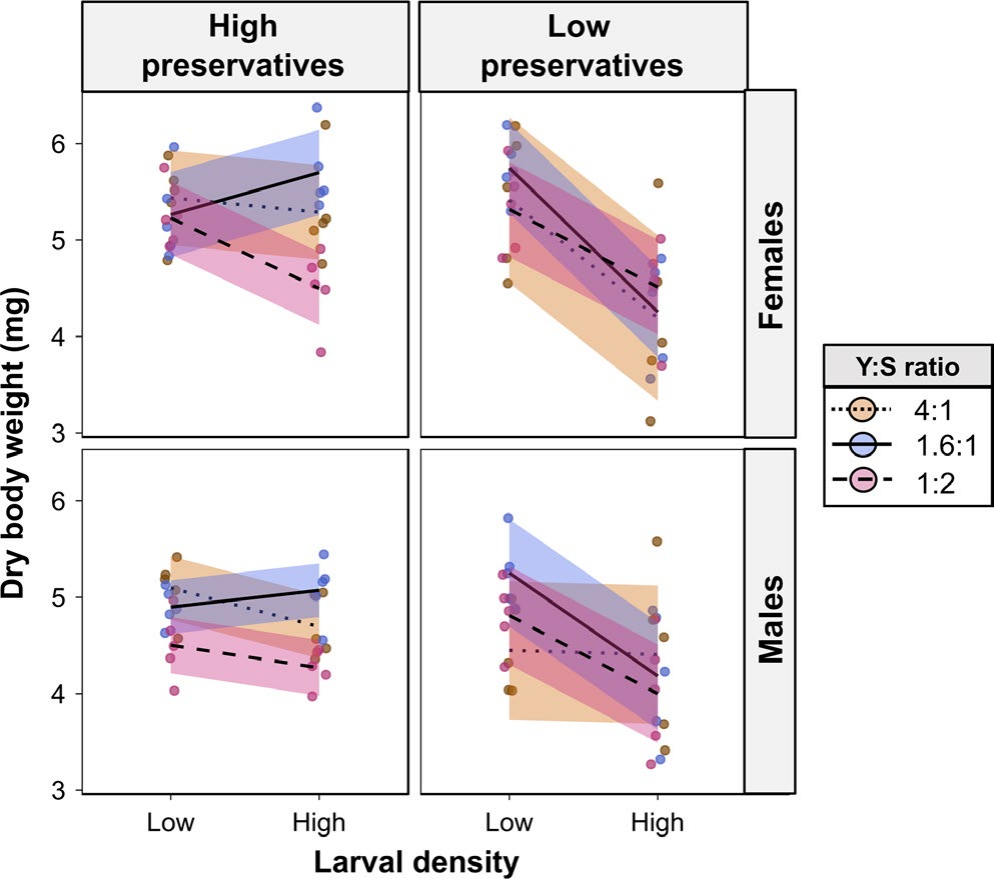
Interactions between larval density, diet, and microbial growth on adult dry weight. Given in mg. Lines were plotted using the ggplot2 package to guide interpretation of the results. Orange—protein‐rich diet (Y:S ratio 4:1); Blue—standard gel‐based diet (Y:S ratio 1.6:1); Magenta—sugar‐rich diet (Y:S ratio 1:2). “High preservatives”—diets with low preservative content where microbial growth was inhibited; “Low preservatives*”*—diets with low preservative content where microbial growth was encouraged. Points were “jittered” horizontally to avoid overlapping. Solid lines were drawn with the “loess” method from the “ggplot2” package to highlight trends in the data

For females, dry body weight decreased from low to high density on all diet compositions when preservative content was low. However, when preservative content was high, a decrease of dry body weight was only observed in sugar‐rich diet, whereas dry body weight remained constant or even increased slightly from low to high larval density in protein‐rich and standard diets (Figure 3), respectively.

For males, dry body weight also decreased from low to high density when preservative content was low, but only for standard and sugar‐rich diets (Figure 3). Dry body weight remained constant between low and high density when on protein‐rich diet with low preservative content (Figure 3). However, when preservative content was high, male dry body weight increased slightly from low to high larval density in standard diet, but decreased slightly in protein‐ and sugar‐rich diets (Figure 3).

### 3.4 Female, but not male body energetic reserves are modulated by the interaction between larval density, diet composition, and preservative content

**Figure 4:**
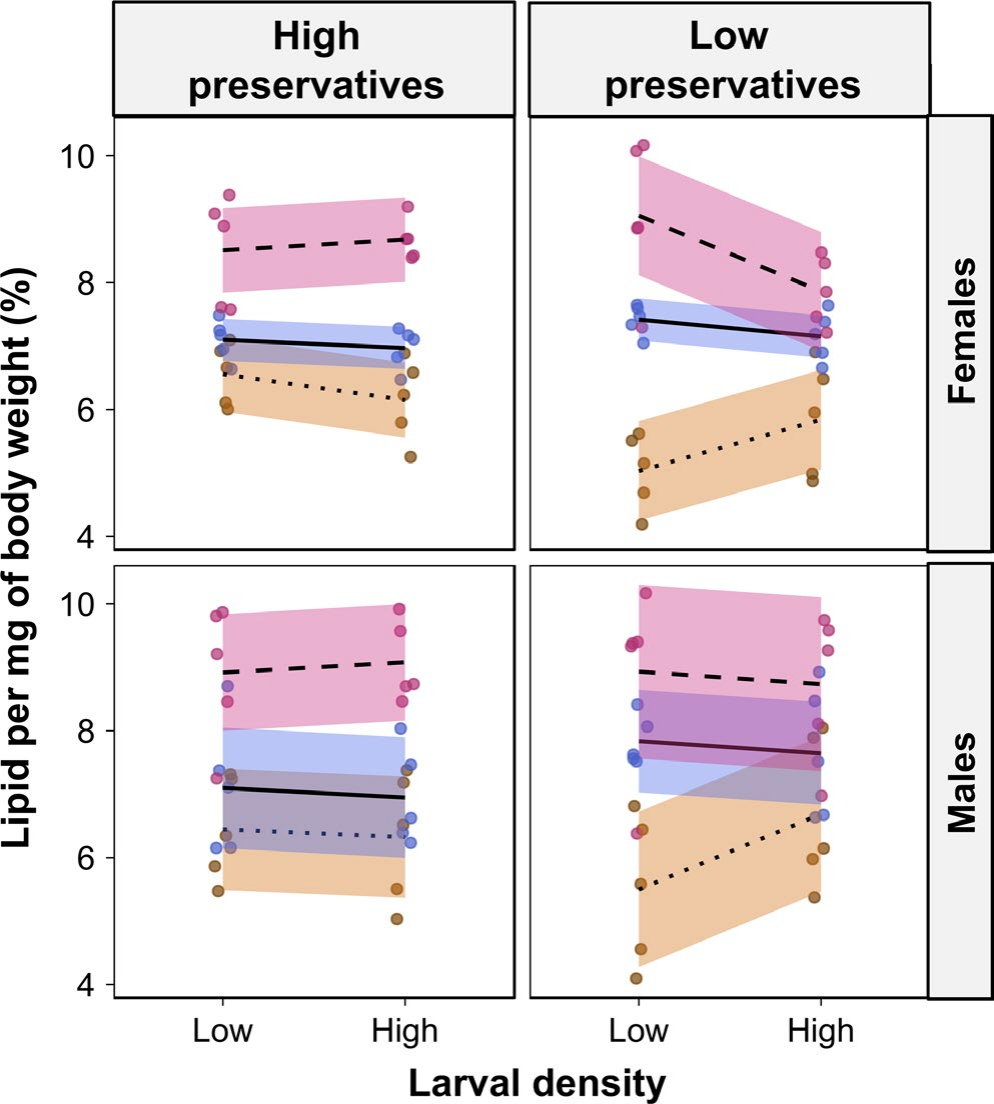
Interactions between larval density, diet, and microbial growth on adult energetic reserves. Given as % of dry body weight. Lines were plotted using the ggplot2 package to guide interpretation of the results. Orange—protein‐rich diet (Y:S ratio 4:1); Blue—standard gel‐based diet (Y:S ratio 1.6:1); Magenta—sugar‐rich diet (Y:S ratio 1:2). “High preservatives”—diets with low preservative content where microbial growth was inhibited; “Low preservatives*”*—diets with low preservative content where microbial growth was encouraged. Points were “jittered” horizontally to avoid overlapping. Solid lines were drawn with the “loess” method from the “ggplot2” package to highlight trends in the data

In females, there was a statistically significant three‐way interaction between larval density, diet composition, and preservative treatment on lipid storage as a percentage of dry mass (*F*_2,48_ = 5.540, *p* = 0.006, Table S6). Overall, in females, lipid storage was higher in sugar‐rich diet, intermediate in standard diet and lower in protein‐rich diet. Effects of larval density on lipid storage in females varied with preservative content; when preservative content was high, there was a slight decrease in lipid storage from low to high larval density in protein‐rich diets, but no difference in standard and sugar‐rich diets (Figure 4). However, when preservative content was low, there was a sharp increase in lipid storage from low to high larval density in protein‐rich diet, which was absent in standard diet and negative in sugar‐rich diet (Figure 4). The two‐way interactions between larval density and preservative treatment, as well as the main effects of diet composition, were also statistically significant in the model for female lipid storage (Table S6). The inclusion of nine outliers in the data resulted in the three‐way interaction to become borderline nonsignificant (*p* = 0.063, Table S7), the two‐way interaction between diet composition and preservative treatment to become borderline nonsignificant (*p* = 0.0503, Table S7), and the main effects of microbial growth and the interaction between larval density and diet composition to become weakly statistically significant (*p* = 0.044 and *p* = 0.019), respectively; Table S7).

In males, only diet composition had a statistically significant effect on the percentage of lipid stored (*F*_2,56_ = 33.558, *p* < 0.001, Table S6), whereby males in sugar‐rich diet had higher percentage of lipid stored, intermediate percentage in standard diet, and lower percentage in protein‐rich diet (Figure 4). Neither the three‐way interaction between larval density, diet composition, and preservative treatment, nor the two‐way interactions between larval density and diet composition, diet composition and preservative treatment, and preservative treatment and larval density influenced male percentage of lipid stored (Table S6). The inclusion of three outliers had no qualitative effects on the analysis (Table S7).

## 4 DISCUSSION

In this study, we demonstrated how larval density, diet composition, and preservative content in the diet (which influence microbial growth) interact to shape larval development in the tephritid fruit fly *B. tryoni*. We found a significant three‐way interaction between larval density, diet composition, and preservative treatment for pupal (Figure 2) and adult weights (Figure 3), as well as for female (but not male) lipid storage (Figure 4). Because preservative treatment had notable effects on dietary microbial growth in our experiments (see Figure S1), we henceforth refer to our findings related to preservative treatment in terms of microbial growth. From our results, general trends can be deduced. First, our results showed that diet composition was a major factor influencing pupal weight as well as lipid storage, although diet‐dependent effects were strongly modulated by microbial growth. For example, protein‐rich and standard larval diets generated heavier pupae relative to sugar‐rich diet when microbial growth was inhibited, but this relationship was reversed when microbial growth was encouraged. These findings corroborate our predictions 3 and 4 (see Table 1) and support our hypotheses that high larval density can induce nutrient‐poor environments and that protein is essential for adequate larval development. Males and females from sugar‐rich diets stored more lipid in accordance to our predictions 5 and 6, which could suggest that *B. tryoni* larvae reared on sugar‐rich diets express an obese‐like phenotype with higher lipid storage as seen in *Drosophila melanogaster* (Musselman et al., 2011; Na et al., 2013; Rovenko et al., 2015). In contrast, the Mediterranean fruit fly (Tephritidae: *Ceratitis captata*) decreased lipid storage when larvae developed in sugar‐rich diets (Nestel & Nemny‐Lavy, 2008). In sugar‐rich diets, female lipid storage decreased from low to high larval density, but in protein‐rich diets increased from low to high larval density. If increased lipid storage is detrimental to female *B. tryoni* as it is for *D. melanogaster* (Musselman et al., 2011; Na et al., 2013; Rovenko et al., 2015), then microbial growth and larval density could act in synergy to reduce the negative effects of sugar‐rich diet (see below). Further studies are nonetheless needed to provide a better understanding of the relationship between fitness and lipid storage in tephritid fruit flies. Our results also showed that, in general, high larval density had a negative effect on pupal and adult weights, which are in agreement with our predictions 1 and 2 as well as previous studies of diverse insect taxa (Bauerfeind & Fischer, 2005; Blanckenhorn, 1998; Lyimo, Takken, & Koella, 1992; Morimoto et al., 2016; Morimoto, Ponton, Tychsen, Cassar, & Wigby, 2017; Wertheim et al., 2002). This confirms our hypothesis that high larval density is costly for individuals (Table 1). However, this effect was not observed in standard diet when microbial growth was inhibited or in protein‐rich diets when microbial growth was encouraged (see Figure 3). Importantly, our results suggest that the potential surplus of protein availability arising from microbial growth in the diet could be insufficient to overcome the negative effects of high larval density, given that pupal and adult weights were generally lower in high larval density treatments independently of microbial growth. Thus, although microbes may serve as food (Fitt & O'Brien, 1985) and potentially as a source of limiting nutrients in a crowded larval developmental environment (Klepsatel et al., 2018), there seem to exist a more complex relationship between larval feeding and microbial growth that warrants further investigation (see Discussion below on animal–microbe competition).

Our findings can provide insights into the ecological factors that modulate larval development. For instance, our findings corroborated our predictions (Table 1) and showed an overall tendency for larvae developing in sugar‐rich diet in high larval density without microbial growth to be lighter (pupae and adults) but fatter (females) than those foraging in protein‐rich or standard diets in high density with inhibited microbial growth. Previous studies in other species corroborate these effects of sugar‐rich diets in larval development (see for instance Matavelli et al., 2015; Silva‐Soares, Nogueira‐Alves, Beldade, & Mirth, 2017; Zucoloto, 1987,1991). From our results, we can predict that when larvae encounter a sugar‐rich diet (or an unfavorable diet more generally), they would be more likely to disperse in search of diets with higher nutritional value, hence resulting in smaller larval aggregates in unfavorable diets. Given that the nutritional composition of fruits varies across strata within fruits (spatial variation) as well as during the ripening process (temporal variation) (Janzen, 1977; Matavelli et al., 2015), larvae could migrate to and aggregate in different strata within a fruit and potentially (although less likely) move from one fruit to another in more nutritious ripening conditions. Spatial aggregation is known to occur across insect species (Taylor, 1961), including *B. tryoni* (Morimoto et al., 2018)*;* larval movement between fruits remains subject of further investigation. Diet‐dependent larval aggregation can influence larval development rate because *B. tryoni* larvae can—like many other species (see Taylor, 1961; Taylor et al., 1978)—benefit from larval aggregation (Morimoto et al., 2018) (see also Discussion on the “Allee effect” below). A recent study has shown that *B. tryoni* larvae tend to aggregate in nutrient‐rich diets that support increased larval growth, whereas dispersal is favoured in nutrient‐poor diets where high larval aggregation incurs a significant cost to larval growth (Morimoto et al., 2018). Similarly, based on our data, we hypothesized that larvae should forage preferentially on microbe‐free protein‐rich diets instead of protein‐rich diets with microbes, because microbial growth in protein‐rich diets has negative effects of larval development and adult traits. This can help understand female oviposition preferences for ripe fruits, in which microbial growth and protein content of the substrate are relatively lower compared with unripe (low protein, low microbial growth) and rotting fruits (high protein, high microbial growth) (Clarke et al., 2011; Rattanapun, Amornsak, & Clarke, 2009).

In nature, *B. tryoni* larvae—as larvae of most Tephritidae—develop in dynamic environments characterized by larval aggregation, microbial growth accompanying fruit ripening and decaying, as well as patches of food sources with varying nutritional compositions within fruits in one generation and between fruits across generations (Clarke et al., 2011; Deutscher, Reynolds, & Chapman, 2016; Drew, 1988; Drew & Lloyd, 1989; Fitt & O'Brien, 1985). Our findings provide the first direct attempt to understand the complex network of interactions among these factors that determine the quality of the larval development in this species. Our results demonstrate a general negative effect of high larval density on larval development and adult traits. *Bactrocera tryoni* larvae tend to aggregate (Morimoto et al., 2018) and females have evolved mechanisms to mitigate the negative fitness effects of high larval density on their offspring. Adult females decrease egg laying upon encountering substrates already inhabited by larvae (Fitt, 1984). It is still unknown how females modulate oviposition in the presence of larvae but in substrates with different nutritional values. For instance, it will be interesting to know whether high larval density in nutrient‐rich and nutrient‐poor diets have the similar effects on female oviposition, or whether females are able to fine‐tune their oviposition based on both nutritional quality and larval social environment. Another crucial factor underpinning larval development is microbial growth, which our results have shown to be particularly important when larvae are exposed to protein‐poor diets. It may be possible that, when protein‐rich substrates are scarce, adult females modulate their oviposition behavior so as to oviposit in nutrient‐poor but microbial‐rich substrates, thereby facilitating larval development; this hypothesis remains to be tested.

The complexity of the larval developmental environment has been investigated within the theoretical framework of the Allee effect. The Allee effect suggests a positive effect of larval aggregation on fitness traits up to a threshold, after which the costs of larval competition offset benefits (Stephens, Sutherland, & Freckleton, 1999). In this context, Wertheim et al. (2002) manipulated the density of larvae (to simulate different larval aggregations) and the number of adults exposed to a fruit substrate prior to inoculation of larvae to test whether the interaction between ecological factors modulated the strength of the Allee effects on *D. melanogaster* larvae. The authors assessed whether fungal growth in the substrate was affected by exposure of fruit to the larvae and adults, and the implications of fungal growth for larval development. Microbial growth had negative effects on larval development, reducing survival of *D. melanogaster* larvae and size of the emerging adults (see both [Trienens, Keller, & Rohlfs, 2010; Wertheim et al., 2002] for similar results). These results are similar to our findings for the protein‐rich and standard diets for which microbial growth resulted in lighter pupae, although we did not find the same pattern for adult dry weight or lipid storage. Nonetheless, negative effects of microbial growth on larval development have been suggested as evidence for animal–microbe competition for the food substrate. Such competition can lead to the evolution of toxic compounds that decrease larval survival and growth (Rohlfs & Churchill, 2011; Trienens et al., 2010; Trienens & Rohlfs, 2012; Wertheim et al., 2002). Therefore, in our study, it is possible that harmful microbes could have grown in protein‐rich and standard diets, and their presence resulted in negative effects for the larvae until pupation in these environments. Sugar‐rich diets, on the other hand, might have favoured the growth of different—potentially less harmful—microbes that could also serve as an additional source of amino acids for the larvae and promote larval development (as in Drew et al., 1983; Fitt & O'Brien, 1985). This could explain our finding that pupae were heavier in sugar‐rich diet when microbial growth was encouraged. Our finding of positive effects of microbial growth in sugar‐rich diets are in agreement with some studies suggesting a positive effect of microbial growth on larval development due to the changes in diet composition caused by microbes (Matavelli et al., 2015) as well as studies showing that microbes can be a direct source of amino acids (Drew et al., 1983; Fitt & O'Brien, 1985). It is also possible that some diets allow beneficial microbes from the larvae to grow and serve as food (“self reinoculation”) while other diets do not allow this process. If this is true, we would expect some diets to have microbial profiles that are more similar to the larvae microbial community than other diets (Chandler, Lang, Bhatnagar, Eisen, & Kopp, 2011) It will be important for future studies to investigate the microbial profile of larval diets with different nutrient compositions because it will provide detailed information of the types of microbes, the potential strength of animal‐microbe competition for the food substrate, and their impact on larval development.

The present study suggests that microbial growth can influence effects of protein‐rich and sugar‐rich diets in ways that minimize the diet‐dependent expression of fitness‐related traits; for instance, for larvae reared on protein‐rich diets microbial growth led to decreased pupal weight and increased female lipid storage whereas for larvae reared on sugar‐rich diets microbial growth led to increased pupal weight and decreased lipid storage. Previous studies have not incorporated the combined effects of diet composition, larval density, and microbial growth, and the present study illustrates the additional insights that can be gained by incorporating such complexity in experimental design. More studies are needed for a better understanding of how diverse ecological factors affect larval foraging behavior and developmental rate of holometabolous insects.

## 5 CONCLUSION

We found a strong interaction between larval density, diet composition, and microbial growth (through the manipulation of preservative content in the diet) on pupal and adult traits of *B. tryoni*, highlighting the importance of multiple ecological factors in shaping the developmental environment of insect larvae. Given that the developmental environment modulates the expression of life history traits in other invertebrates (Ireland & Turner, 2006; Tavares, Pestana, Rocha, Schiavone, & Guillermo‐Ferreira, 2018) and vertebrates (including humans) (Gilbert & Epel, 2009; Gluckman & Hanson, 2006), studies that address how ecology modulates the development of life history traits can help us gain insights into how developmental ecology influence evolutionary processes and adaptions across the animal kingdom (Gilbert, 2001).

## CONFLICT OF INTERESTS

None to declare.

## AUTHORS' CONTRIBUTIONS

JM designed the experiment. JM, BN, FP, and ATT collected data. All authors analyzed the data, provided inputs into the writing of the manuscript, and approved the final version.

### DATA ACCESSIBILITY

The raw data used in the study were deposited in Dryad: https://doi.org/10.5061/dryad.qr54869.
