# Supplementary material for: Interactions between ecological factors in the developmental environment modulate pupal and adult traits in a polyphagous fly
Source: Ecol Evol. 2019 Apr 26;9(11):6342–52. doi: 10.1002/ece3.5206 (PMC6580268; doi:10.1002/ece3.5206)
Supplement: Supplementary file 2 [file ECE3-9-6342-s002.pdf]

Supplementary Information: ‘Interactions between ecological factors in the developmental environment modulate pupal and adult traits in a polyphagous fly’

Authors: Binh Nguyen[1], Fleur Ponton[1], Anh Than[1,2], Phillip W. Taylor[1], Toni Chapman[3], Juliano Morimoto[1*]

Running title: Diet, density, and microbe effects on fitness

Author’s affiliations:

1 – Department of Biological Sciences, Macquarie University, NSW 2109, Australia

2 – Department of Entomology, Vietnam National University of Agriculture, Hanoi, Vietnam

3 – The Elizabeth Macarthur Agricultural Institute, New South Wales Department of Primary Industries, NSW 2568, Australia

*To whom correspondence should be addressed:

Juliano Morimoto

Address: Department of Biological Sciences, Macquarie University, NSW 2109, Australia

E-mail: juliano.morimoto@mq.edu.au

Data accessibility

The raw data used in the study was deposited in Dryad: doi:10.5061/dryad.qr54869

Authors’ contributions

JM designed the experiment. JM, BN, FP, ATT collected the data. All authors analysed the data, provided inputs into the writing of the manuscript, and approved the submitted version.

Supplementary Figures

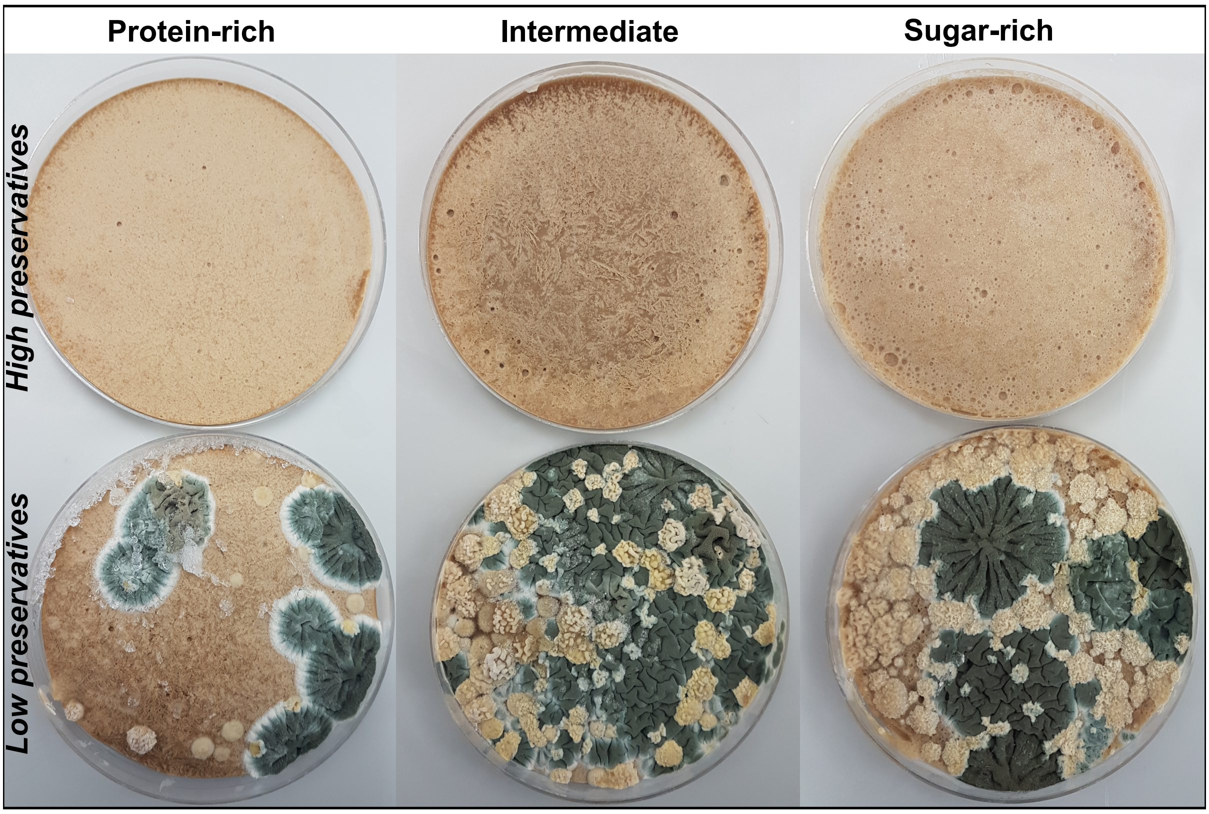

Fig S1 – The microbial growth stimulated by the preservative treatment used in our study. Control diets (i.e., no larvae) with high preservatives content and low preservatives content (high microbe growth) after 6 days incubation at 25 ± 0.5^{o}C and 65 ± 5% relative humidity. Microbe growth as observed 36-48h after the plates wer

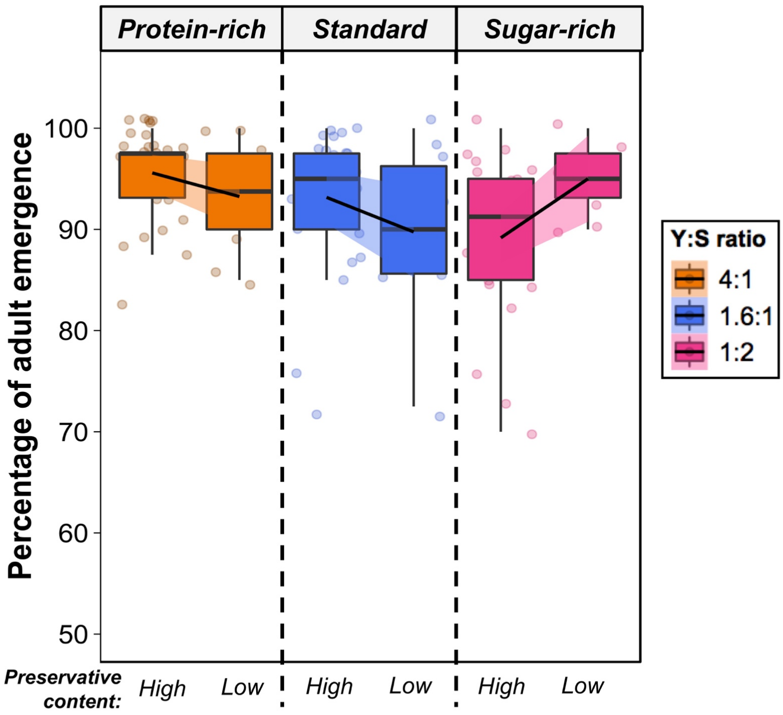

Fig S2 – Diet and preservative content interact to determine the percentage of adult emergence. Given in %. Orange – protein-rich diet (Y:S ratio 4:1); Blue – standard gel-based diet (Y:S ratio 1.6:1); Magenta – sugar-rich diet (Y:S ratio 1:2). Note that because larval density had no statistically significant effect, we omitted this factor from the graph, and lines connect the trend between treatments with high and low preservative contents. Points were "jittered" to avoid overlapping. Solid lines were drawn with the 'loess' method from the 'ggplot2' package to highlight trends in the data.

Supplementary Tables and Statistical Analysis

Table S1 – Diet recipes.

Ingredient	Protein-rich diet	Standard diet	Sugar-rich diet
Brewer's Yeast (g)	260.6	204	108.6
Sugar (g)	65.2	121.8	217.2
Agar(g)	10	10	10
Citric Acid (g)	23	23	23
Nipagin (g)	2	2	2
Sodium benzoate (g)	2	2	2
Wheat Germ Oil (ml)	2	2	2
MiliQ Water (ml)	1000	1000	1000

Model selection

Model selection has been used in complex statistical models with high-level interaction terms (e.g., three-interactions), such as those used in our study. Because we adopted a ‘full model approach’, it is possible that our high-level interaction terms decrease the power of our analysis, thereby decreasing our ability to detect statistically significant effects. To overcome this, we re-ran our analysis while performing backwards model selection using the ‘step’ function in R. Model selection was based on AIC values. Table S1 shows the step-by-step model simplification, the final models, and their AIC scores.

Table S2 – Step-by-step model selection for models used in this study.

Model	AIC
Pupal weight	
Final Model: Larval density * Diet composition * Preservative treatment	157.60
Adult emergence[∂]	
Full Model: Larval density * Diet composition * Preservative treatment	-307.48
Larval density + Diet composition + Preservative treatment + Larval density * Diet composition + Larval density * Preservative treatment + Diet composition * Preservative treatment	-310.48
Larval density + Diet composition + Preservative treatment + Larval density * Preservative treatment + Diet composition + Preservative treatment	-313.51
Larval density + Diet composition + Preservative treatment + Diet treatment * Preservative treatment	-315.37
Final Model: Diet composition + Preservative treatment + Diet treatment * Preservative treatment	-317.30
Body weight (Female)	
Final Model: Larval density * Diet composition * Preservative treatment	106.12
Body weight (Male)	
Final Model: Larval density * Diet composition * Preservative treatment	89.81
Lipid storage (Female)[∂]	
Final Model: Larval density * Diet composition * Preservative treatment	-425.78
Lipid storage (Male)[∂]	
Full Model: Larval density * Diet composition * Preservative treatment	-366.80
Larval density + Diet composition + Preservative treatment + Larval density * Diet composition + Larval density * Preservative treatment + Diet composition * Preservative treatment	-368.5
Larval density + Diet composition + Preservative treatment + Larval density * Preservative treatment + Diet composition * Preservative treatment	-370.96

Larval density + Diet composition + Preservative treatment + Diet composition * Preservative treatment	-372.57
Diet composition + Preservative treatment + Diet composition * Preservative treatment	-374.35
Diet composition + Preservative treatment	-375.11
Final Model: Diet composition	-376.99

Final models were selected based on AIC values. $^{\partial}$- Previous ‘quasibinomial’ models converted to linear models to allow for model selection based on AIC values. No qualitative changes on statistical inferences due to model conversion were detected (Table S3-S6).

There were no qualitatively differences between the statistical significance in final models after model selection and our full-model approach presented in the main text (Tables S3-S6). This suggests that, despite retaining two- and three-way interactions, our full-model approach had enough power to detect statistical significance whenever they existed in the data.

Table S3 – Full model outputs and model selection comparison for the analysis of pupal weight. (Table provided in separate Excel file)

Table S4 – Full model outputs and model selection comparison for the analysis of percentage of adult emergence and sex ratio. Note that to perform model selection, the original *quasibinomial* full model had to be converted into linear model, upon which AIC values could be used for model selection. No qualitative differences in statistical significance of factors from this conversion were observed. (Table provided in separate Excel file)

Table S5 – Full model outputs and model selection comparison for the analysis of female and male adult weights. (Table provided in separate Excel file)

Table S6 – Full model outputs and model selection comparison for the analysis of percentage of body lipid stored in adult flies. Note that to perform model selection, the original *quasibinomial* full model had to be converted into linear model, upon which AIC

values could be used for model selection. No qualitative differences in statistical significance of factors from this conversion were observed. (Table provided in separate Excel file)

Table S7 – Full model outputs and model selection comparison for the analysis of percentage of body lipid stored in adult flies with outliers.
